# Supplementary material for: Molecular and spatial transcriptomic classification of midbrain dopamine neurons and their alterations in a LRRK2G2019S model of Parkinson’s disease
Source: bioRxiv. 2024 Dec 22:2024.06.06.597807. Preprint. [Version 4] doi: 10.1101/2024.06.06.597807 (PMC11185743; doi:10.1101/2024.06.06.597807)

bioRxiv preprint doi: https://doi.org/10.1101/2024.06.06.597807; this version posted December 22, 2024. The copyright holder for this preprint (which was not certified by peer review) is the author/funder, who has granted bioRxiv a license to display the preprint in perpetuity. It is made available under aCC-BY 4.0 International license.

Figure S1

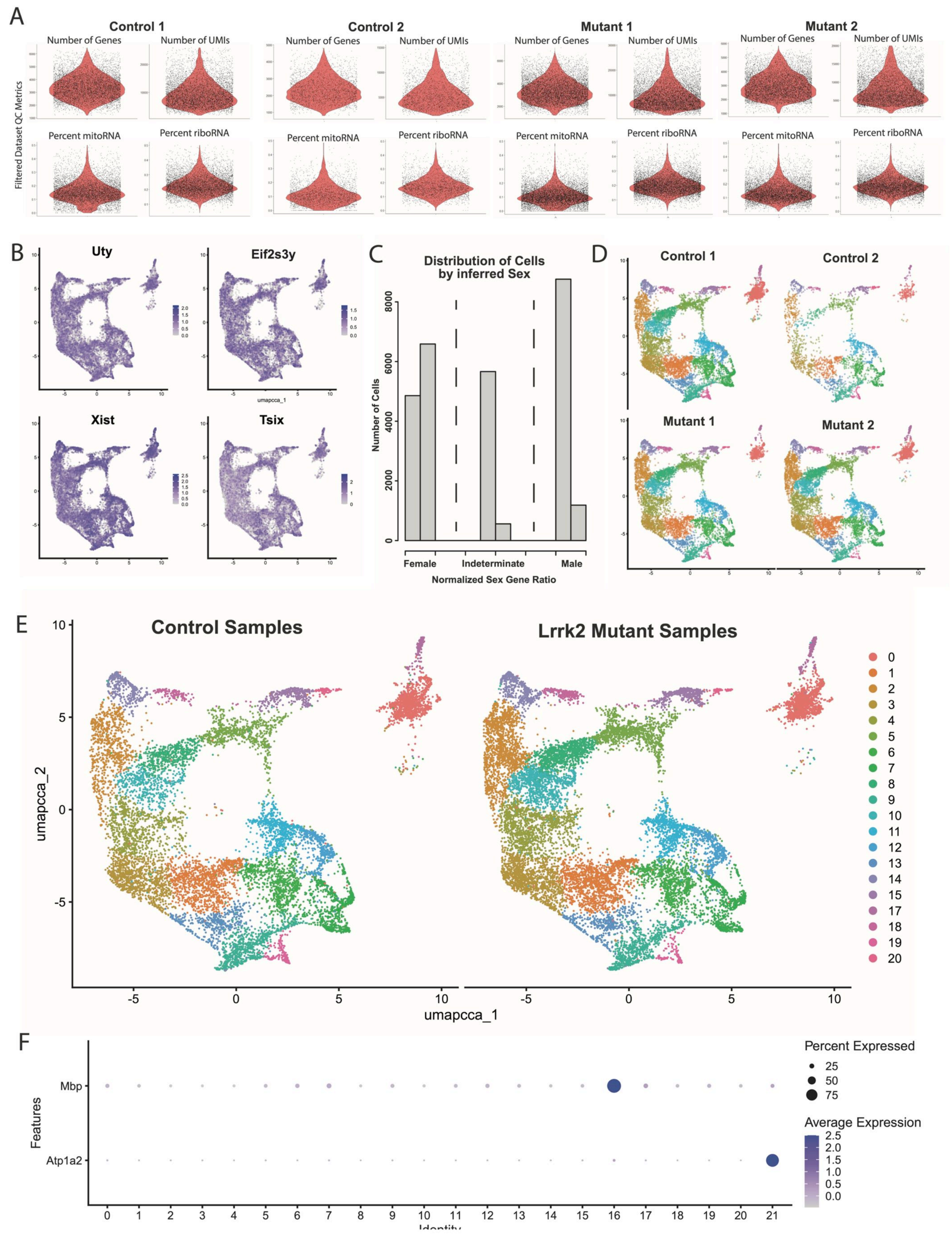

bioRxiv preprint doi: https://doi.org/10.1101/2024.06.06.597807; this version posted December 22, 2024. The copyright holder for this preprint (which was not certified by peer review) is the author/funder, who has granted bioRxiv a license to display the preprint in perpetuity. It is made available under aCC-BY 4.0 International license.

Figure S2

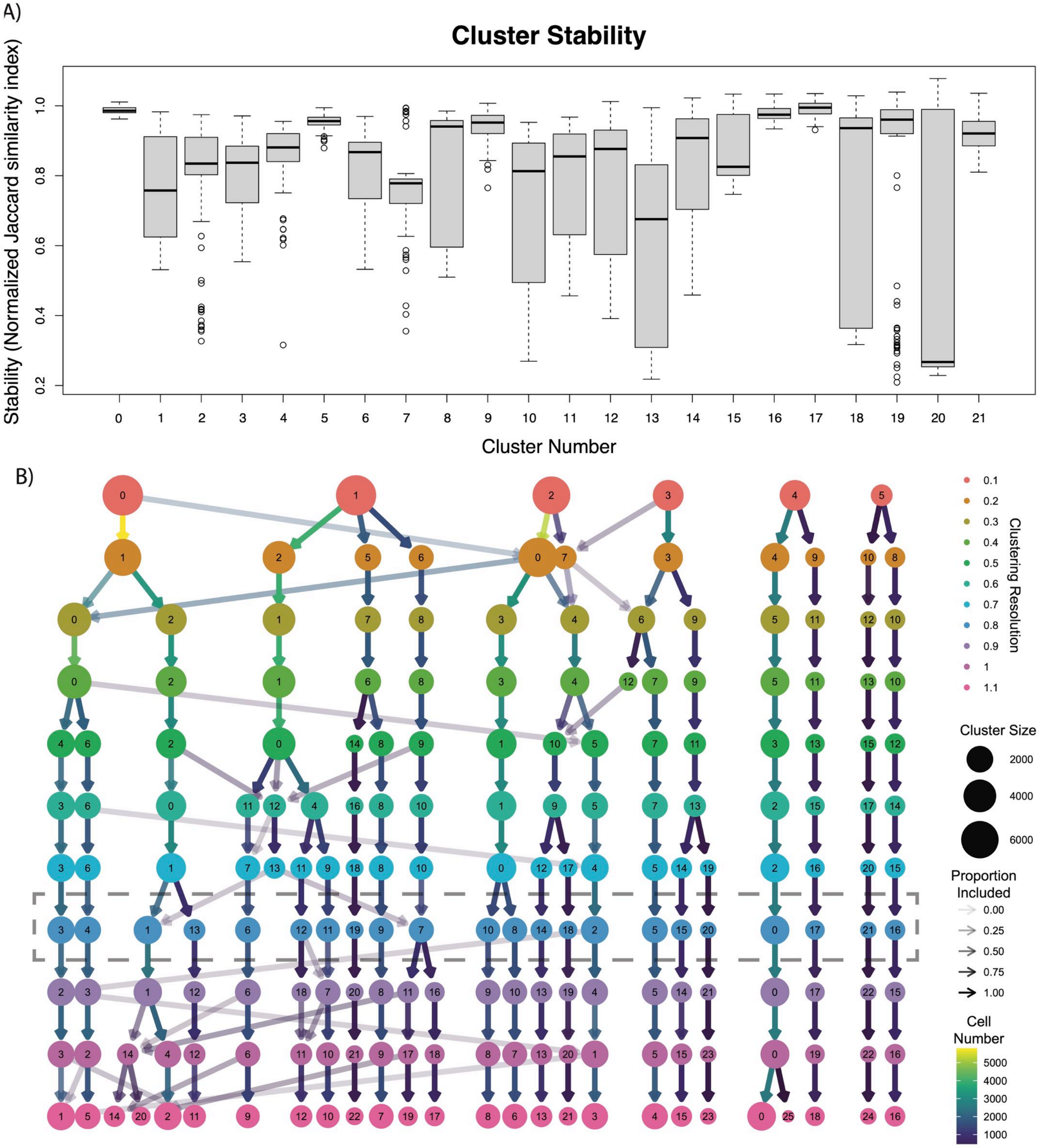

bioRxiv preprint doi: https://doi.org/10.1101/2024.06.06.597807; this version posted December 22, 2024. The copyright holder for this preprint (which was not certified by peer review) is the author/funder, who has granted bioRxiv a license to display the preprint in perpetuity. It is made available under aCC-BY 4.0 International license.

Figure S3

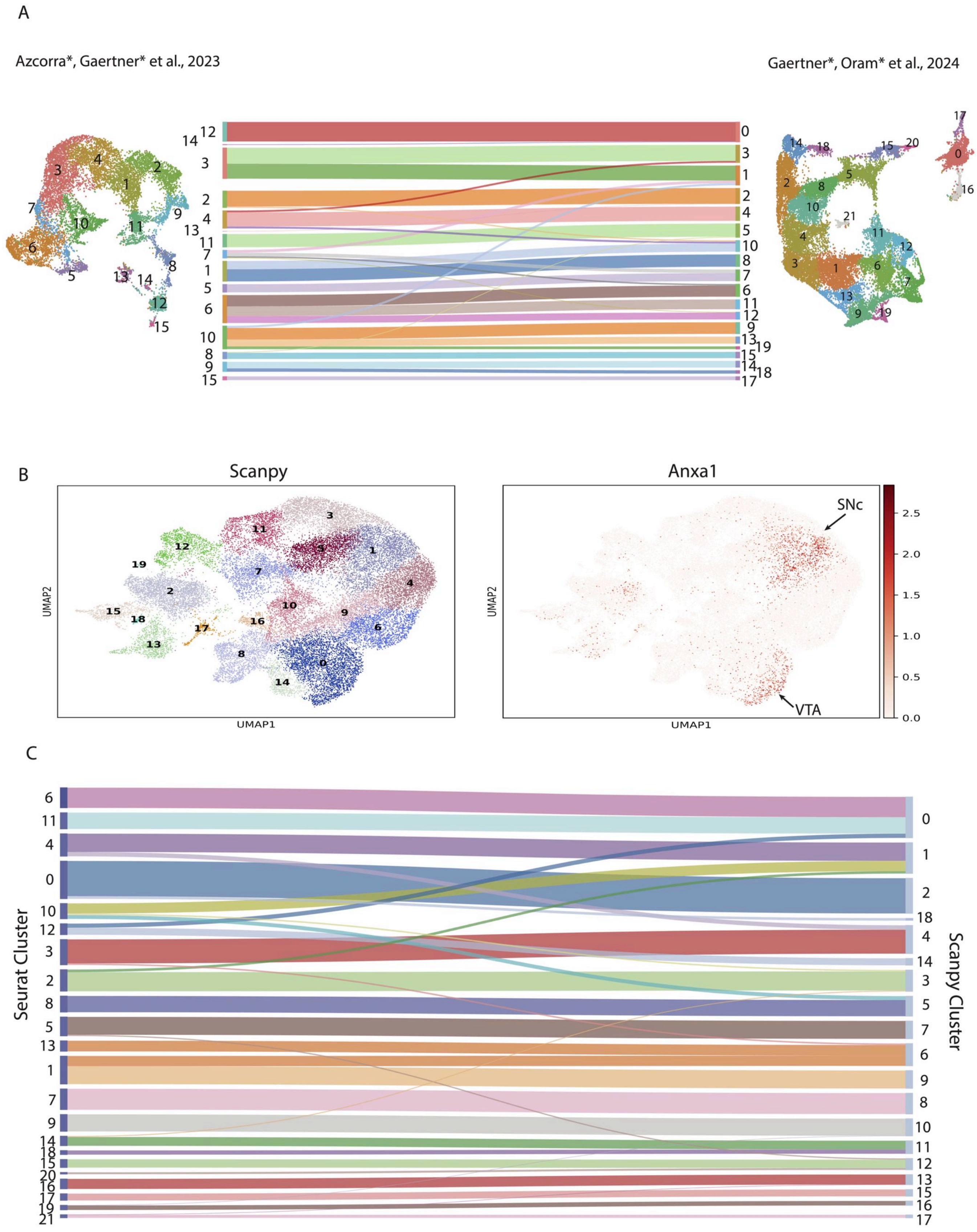

bioRxiv preprint doi: https://doi.org/10.1101/2024.06.06.597807; this version posted December 22, 2024. The copyright holder for this preprint (which was not certified by peer review) is the author/funder, who has granted bioRxiv a license to display the preprint in perpetuity. It is made available under aCC-BY 4.0 International license.

Figure S4

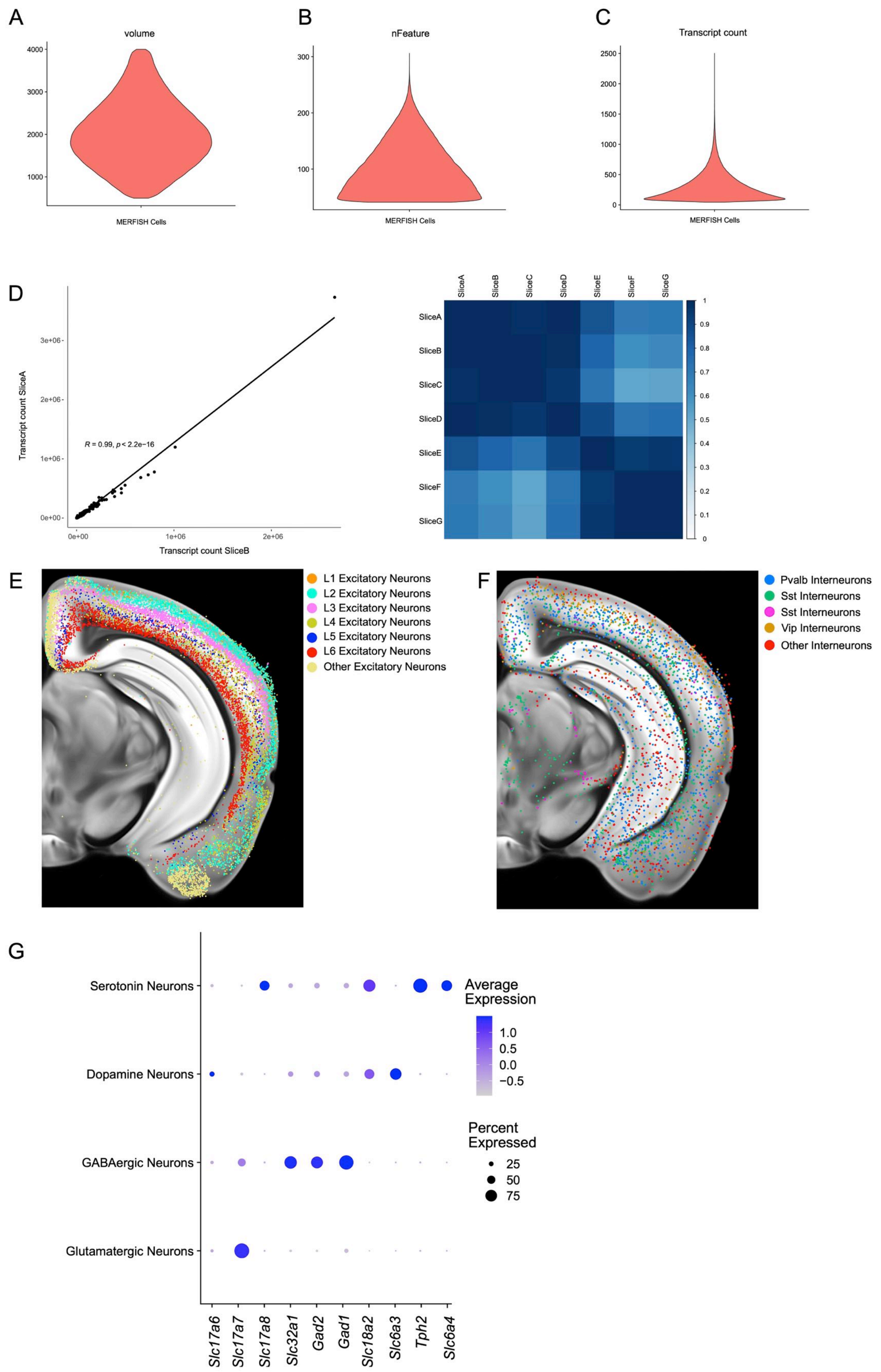

bioRxiv preprint doi: https://doi.org/10.1101/2024.06.06.597807; this version posted December 22, 2024. The copyright holder for this preprint (which was not certified by peer review) is the author/funder, who has granted bioRxiv a license to display the preprint in perpetuity. It is made available under aCC-BY 4.0 International license.

Figure S5

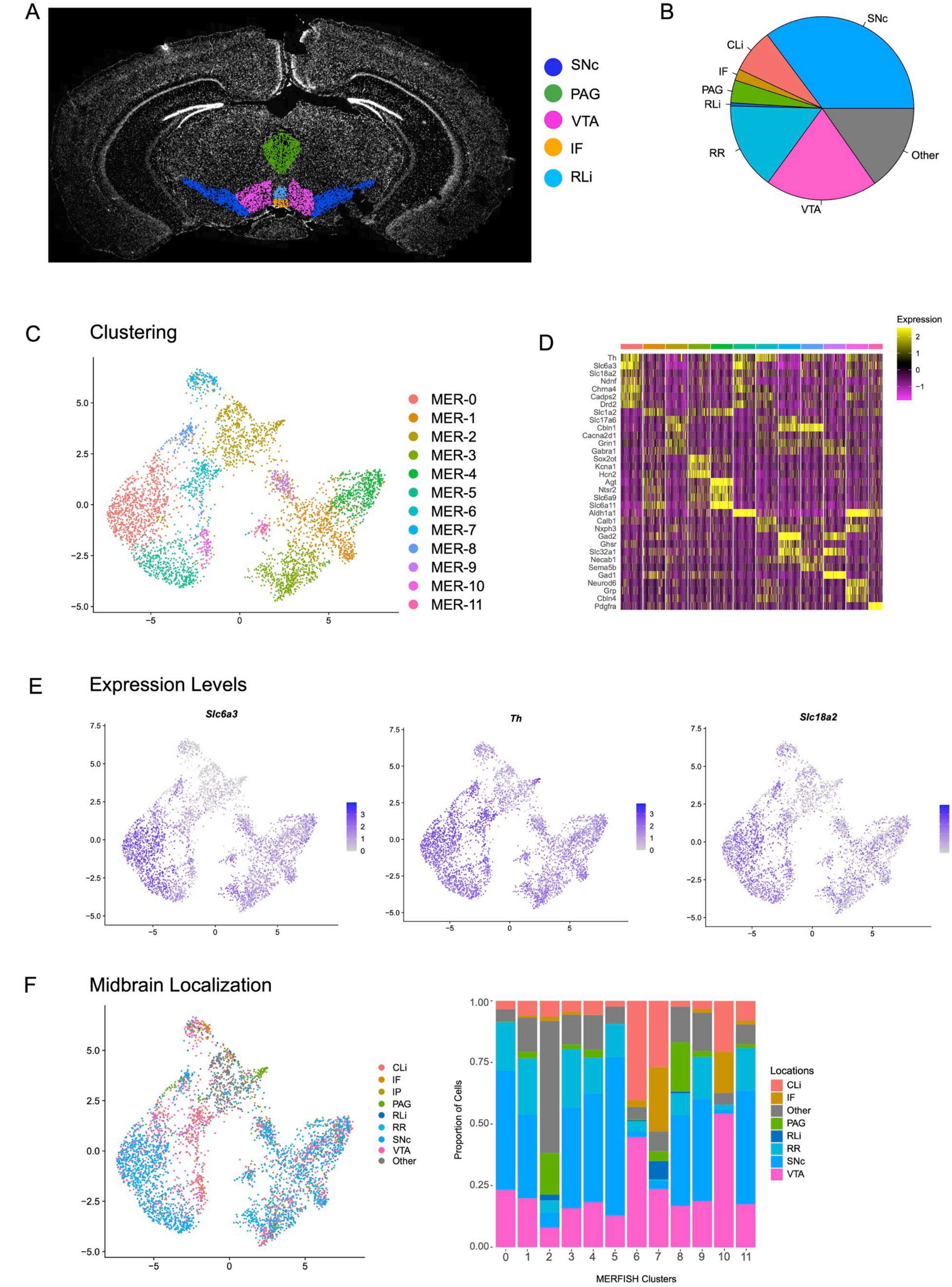

bioRxiv preprint doi: https://doi.org/10.1101/2024.06.06.597807; this version posted December 22, 2024. The copyright holder for this preprint (which was not certified by peer review) is the author/funder, who has granted bioRxiv a license to display the preprint in perpetuity. It is made available under aCC-BY 4.0 International license.

Figure S6

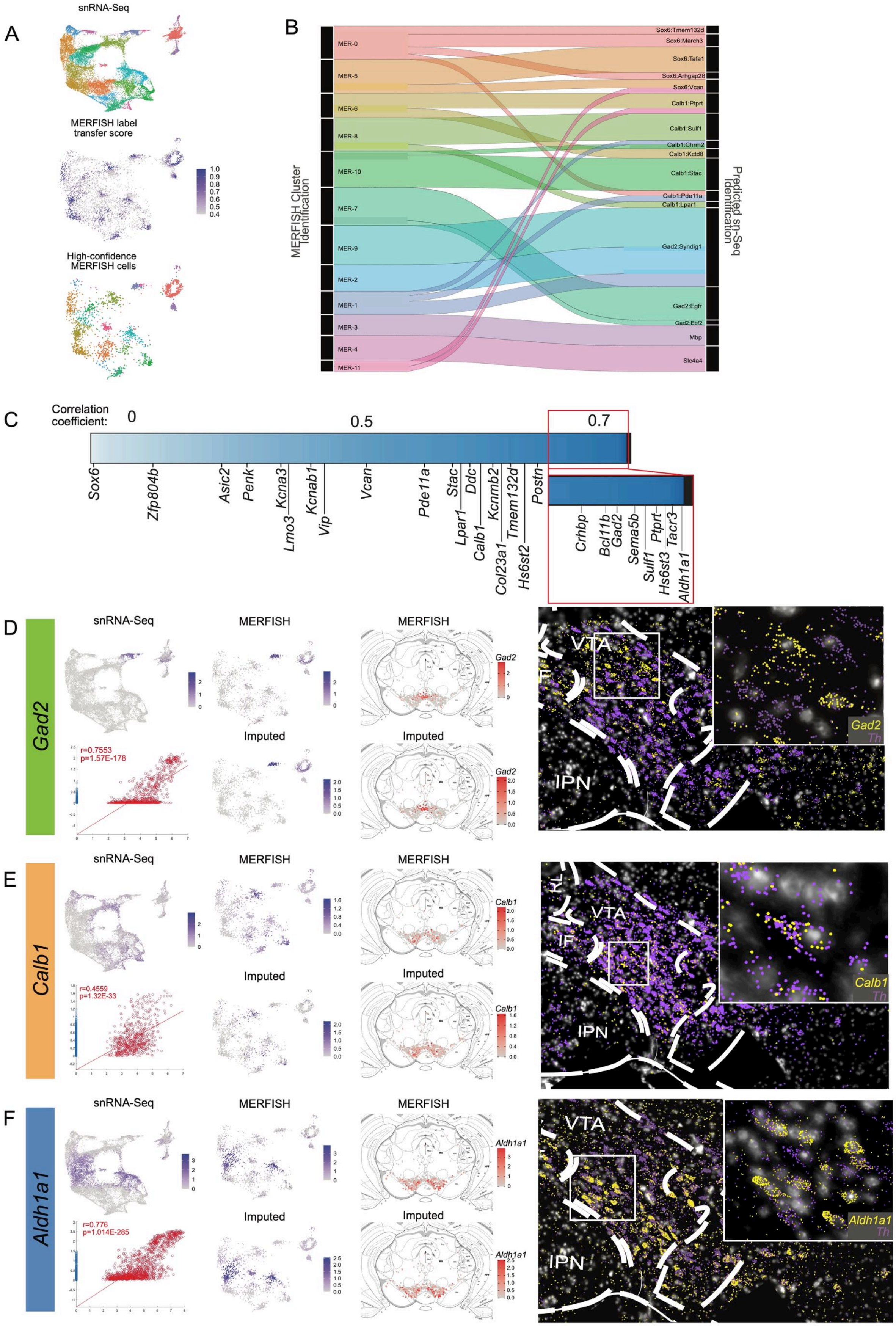

bioRxiv preprint doi: https://doi.org/10.1101/2024.06.06.597807; this version posted December 22, 2024. The copyright holder for this preprint (which was not certified by peer review) is the author/funder, who has granted bioRxiv a license to display the preprint in perpetuity. It is made available under aCC-BY 4.0 International license.

Figure S7

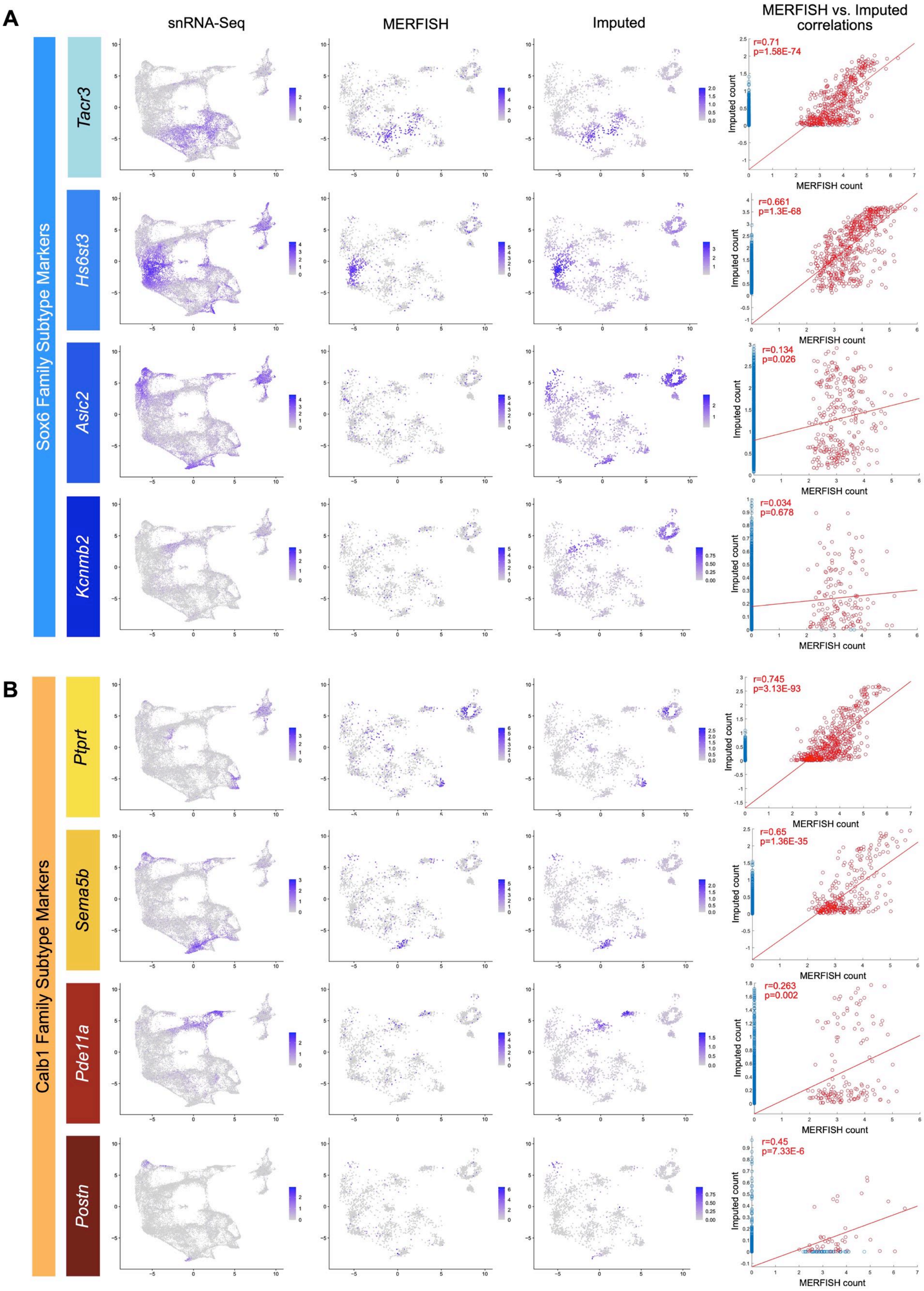

bioRxiv preprint doi: https://doi.org/10.1101/2024.06.06.597807; this version posted December 22, 2024. The copyright holder for this preprint (which was not certified by peer review) is the author/funder, who has granted bioRxiv a license to display the preprint in perpetuity. It is made available under aCC-BY 4.0 International license.

Figure S8

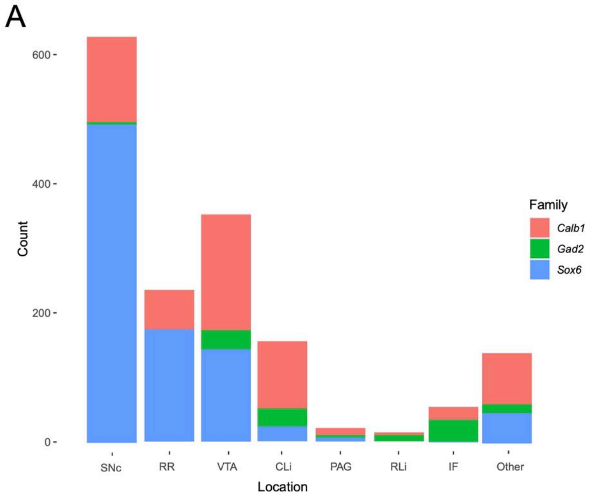

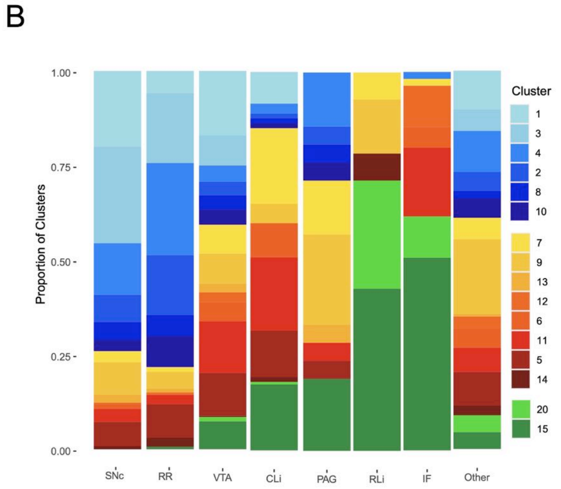

bioRxiv preprint doi: https://doi.org/10.1101/2024.06.06.597807; this version posted December 22, 2024. The copyright holder for this preprint (which was not certified by peer review) is the author/funder, who has granted bioRxiv a license to display the preprint in perpetuity. It is made available under aCC-BY 4.0 International license.

Figure S9

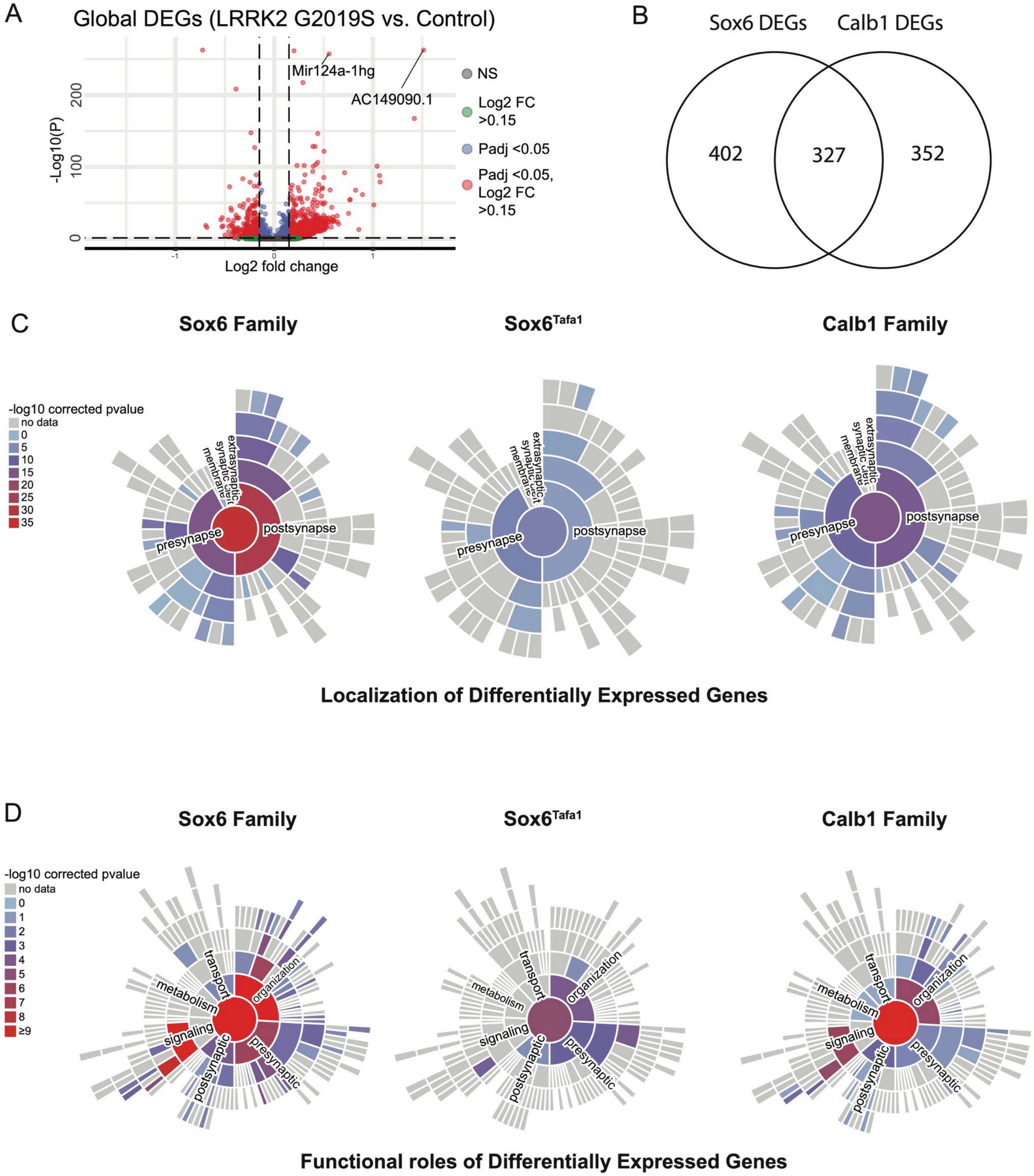

bioRxiv preprint doi: https://doi.org/10.1101/2024.06.06.597807; this version posted December 22, 2024. The copyright holder for this preprint (which was not certified by peer review) is the author/funder, who has granted bioRxiv a license to display the preprint in perpetuity. It is made available under aCC-BY 4.0 International license.

Figure S10

A

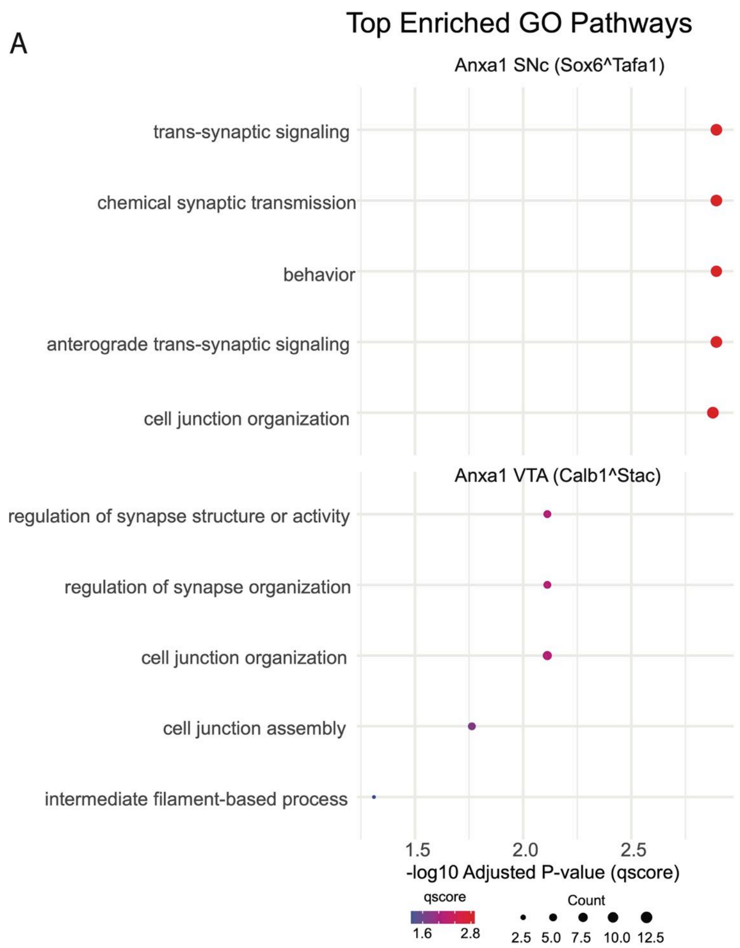

Supplement: Supplement 3 — Figure S1. Quality control for dataset generation. A) Violin plots for each of our 4 samples (post-filtering) for each of the four QC metrics used for filtering our datasets. B) Expression pakerns of sex-specific genes. All clusters are represented by both sexes. C) Histogram of number of cells ploked by male to female gene ratios (scored continuously along the x-axis). Three discrete peaks emerge, representing cells likely originating from either sex or those with indeterminate ratios due to technical drop-off in RNAseq reads. Similar numbers of male and female cells were observed, as represented by the sums of adjacent bins in each of the three marked regions on the x-axis. D) Cluster representation from each individual RNAseq library. All clusters were represented in all samples. E) Cluster representation from pooled control and Lrrk2 mutant samples. Distributions are roughly equivalent between conditions, suggesting no overt change in subtype composition as a function of genotype. F) Dotplot of expression for glial marker genes Mbp and Atp1a2, showing high expression in clusters 16 and 21, respectively, indicating likely doublets of DA neurons and glia. Figure S2. Representations of cluster heterogeneity. A) Cluster stability metrics shown as a box plot for each cluster. Clusters with lower stability contain cells that more easily collapse into other clusters. Outliers (defined as more than 1.5 times the interquartile range (IQR) above the third quartile or below the first quartile) are shown as circles. B) Cluster tree displaying evolution of clusters when calculated at different resolutions. As resolution increases, new levels of heterogeneity emerge, but ultimately become largely stable at higher resolutions. Resolution used for our clustering scheme is highlighted by doked line. Figure S3. Validating snRNA-seq clusters with Python and published DA clusters. A) Comparison of UMAP from Azcorra & Gaertner et al., 202325 and UMAP from Figure 1. A Sankey diagram [file NIHPP2024.06.06.597807v4-supplement-3.pdf]
