# Supplementary material for: Molecular and spatial transcriptomic classification of midbrain dopamine neurons and their alterations in a LRRK2G2019S model of Parkinson’s disease
Source: bioRxiv. 2024 Sep 25:2024.06.06.597807. Preprint. [Version 3] doi: 10.1101/2024.06.06.597807 (PMC11185743; doi:10.1101/2024.06.06.597807)

bioRxiv preprint doi: https://doi.org/10.1101/2024.06.06.597807; this version posted September 25, 2024. The copyright holder for this preprint (which was not certified by peer review) is the author/funder, who has granted bioRxiv a license to display the preprint in perpetuity. It is made available under aCC-BY 4.0 International license.

Supplementary Figure Legends

Figure S1. Quality control for dataset generation. A) Violin plots for each of our 4 samples (post-filtering) for each of the four QC metrics used for filtering our datasets. B) Expression patterns of sex-specific genes. All clusters are represented by both sexes. C) Histogram of number of cells plotted by male to female gene ratios (scored continuously along the x-axis). Three discrete peaks emerge, representing cells likely originating from either sex or those with indeterminate ratios due to technical drop-off in RNAseq reads. Similar numbers of male and female cells were observed, as represented by the sums of adjacent bins in each of the three marked regions on the x-axis. D) Cluster representation from each individual RNAseq library. All clusters were represented in all samples. E) Cluster representation from pooled control and Lrrk2 mutant samples. Distributions are roughly equivalent between conditions, suggesting no overt change in subtype composition as a function of genotype. F) Dotplot of expression for glial marker genes *Mbp* and *Atp1a2*, showing high expression in clusters 16 and 21, respectively, indicating likely doublets of DA neurons and glia.

bioRxiv preprint doi: https://doi.org/10.1101/2024.06.06.597807; this version posted September 25, 2024. The copyright holder for this preprint (which was not certified by peer review) is the author/funder, who has granted bioRxiv a license to display the preprint in perpetuity. It is made available under aCC-BY 4.0 International license.

Figure S2. Representations of cluster heterogeneity. A) Cluster stability metrics shown as a box plot for each cluster. Clusters with lower stability contain cells that more easily collapse into other clusters. Outliers (defined as more than 1.5 times the interquartile range (IQR) above the third quartile or below the first quartile) are shown as circles. B) Cluster tree displaying evolution of clusters when calculated at different resolutions. As resolution increases, new levels of heterogeneity emerge, but ultimately become largely stable at higher resolutions. Resolution used for our clustering scheme is highlighted by dotted line.

Figure S3. Validating snRNA-seq clusters with Python and published DA clusters. A) Comparison of UMAP from Azcorra*,Gaertner* et al., 2023[25] and UMAP from Figure 1. A Sankey diagram was created by mapping nuclei collected in Fig 1 onto the clusters from Azcorra*,Gaertner* et al., 2023[25]. The sankey diagrams only include cell transfers that make up at least 5% of the cluster. B) UMAP representation of clusters using Scanpy package on Python and expression of Anxa1 gene. C) Sankey diagram comparing the clustering of dataset from Figure 1 using Scanpy and Seurat. Note, cells in cluster 19 of the Scanpy plot did not transfer to one cluster in Seurat with >5% of its total cell count.

Figure S4. Quality control metrics for MERFISH experiments. A-C) Volume, number of features (nFeatures) and number of total transcripts counts (nCounts) metrics for each cell identified in MERFISH experiments after quality control. D) Example correlation coefficient between total cellular transcripts detected for each gene across two experiments and a matrix showing experimental similarity. Spatial location of cortical excitatory (E) and inhibitory (F) neurons show distinct layering patterns by MERFISH imaging. G) Relative expression levels of groups of genes associated with synthesis and secretion of glutamate (*Slc17a6, Slc17a7, Slc17a8*), GABA (*Slc32a1, Gad1, Gad2*), dopamine (*Slc6a3, Th*) and serotonin (*Tph2, Slc6a4*) showcase the multilingual nature of the dopaminergic and serotonergic systems.

Figure S5. Clustering of DA neurons identified by MERFISH. A) Schematic showing the manual outlining of anatomical regions of the ventral midbrain. B) Relative proportions of total DA neurons identified by MERFISH distributed throughout the ventral midbrain. C) Subclustering of DA neurons from MERFISH dataset reveals 12 distinct subclusters. D) Heatmap showing relative expression of genes associated with

bioRxiv preprint doi: https://doi.org/10.1101/2024.06.06.597807; this version posted September 25, 2024. The copyright holder for this preprint (which was not certified by peer review) is the author/funder, who has granted bioRxiv a license to display the preprint in perpetuity. It is made available under aCC-BY 4.0 International license.

dopaminergic character and the top three differentially expressed genes within each cluster identified in c). E) Relative expression of genes associated with dopaminergic character (*Slc6a3*, *Th*, *Slc18a2*) distributed across DA clusters. F) Midbrain location of clusters identified by MERFISH using the manual outlining described in A).

Figure S6. Integrating MERFISH with snRNA-seq: A) Overview of MERFISH integration with snRNA-seq dataset (top). snRNA-seq UMAP space and cluster identification were transferred to MERFISH cells by identifying mutual nearest neighbors (MNN) and assigning a score based on the overlap of neighbor cells that share the same labelling (middle). High confidence MERFISH cells (bottom) were identified as those that showed a cell similarity score >0.5 (2,298 of 4,399 original DA neurons). B) Sankey plot showing the proportion of cells from MERFISH clustering identities that make up the snRNA-seq clustering identities. Flow chart only includes cell transfers that make up at least 10% of the snRNA-Seq cluster. C-E) comparison of the transcript detection in snRNA-seq dataset, MERFISH dataset and imputed gene expression (Gad2, Calb1 and Aldh1a1 - left). Spatial location of normalized transcript counts and imputed data show similar distributions (middle). Example representations of individual transcripts detected by MERFISH in the VTA for each gene (right and inset).

Figure S7. A) Anatomical distribution of individual DA neurons by cluster family. B) Relative proportions of DA cluster subtypes by anatomical region.

Figure S8. Analysis of synaptic localizations and functional roles of differentially expressed genes using SynGO. A) Venn diagram of differentially expressed genes (DEGs) between Sox6 and Calb1 families. B) Mapping of pre- and post-synaptic genes found among significant DEGs in Lrrk2^{G2019S} mutants compared to controls for Sox6 family, Sox6^{Tafa1}, and Calb1 family of clusters. Bars are colored according to relative association with each cellular compartment. C) Functional roles of synaptic DEGs in each population in Lrrk2^{G2019S} mutants compared to controls.

bioRxiv preprint doi: https://doi.org/10.1101/2024.06.06.597807; this version posted September 25, 2024. The copyright holder for this preprint (which was not certified by peer review) is the author/funder, who has granted bioRxiv a license to display the preprint in perpetuity. It is made available under aCC-BY 4.0 International license.

Figure S1

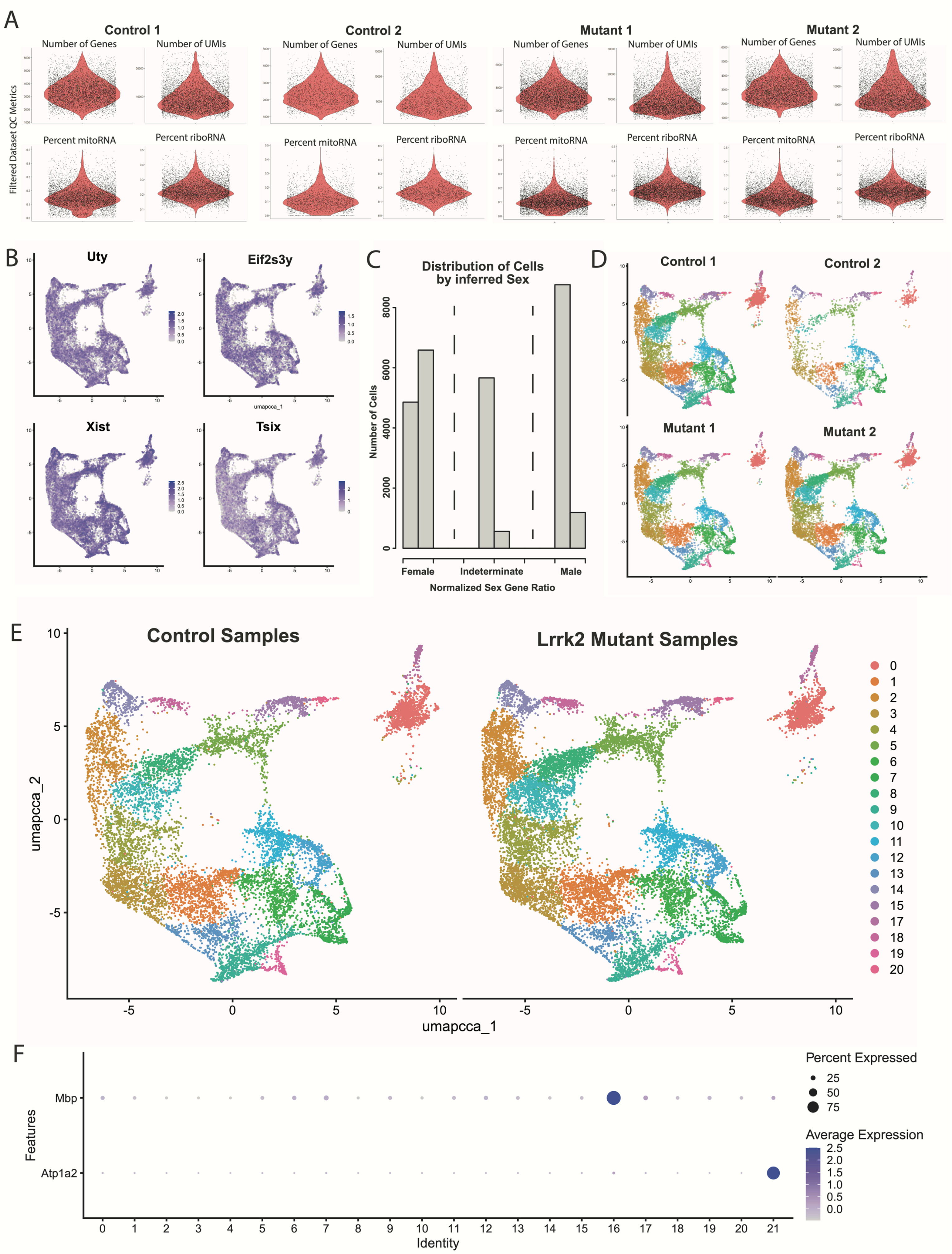

bioRxiv preprint doi: https://doi.org/10.1101/2024.06.06.597807; this version posted September 25, 2024. The copyright holder for this preprint (which was not certified by peer review) is the author/funder, who has granted bioRxiv a license to display the preprint in perpetuity. It is made available under aCC-BY 4.0 International license.

Figure S2

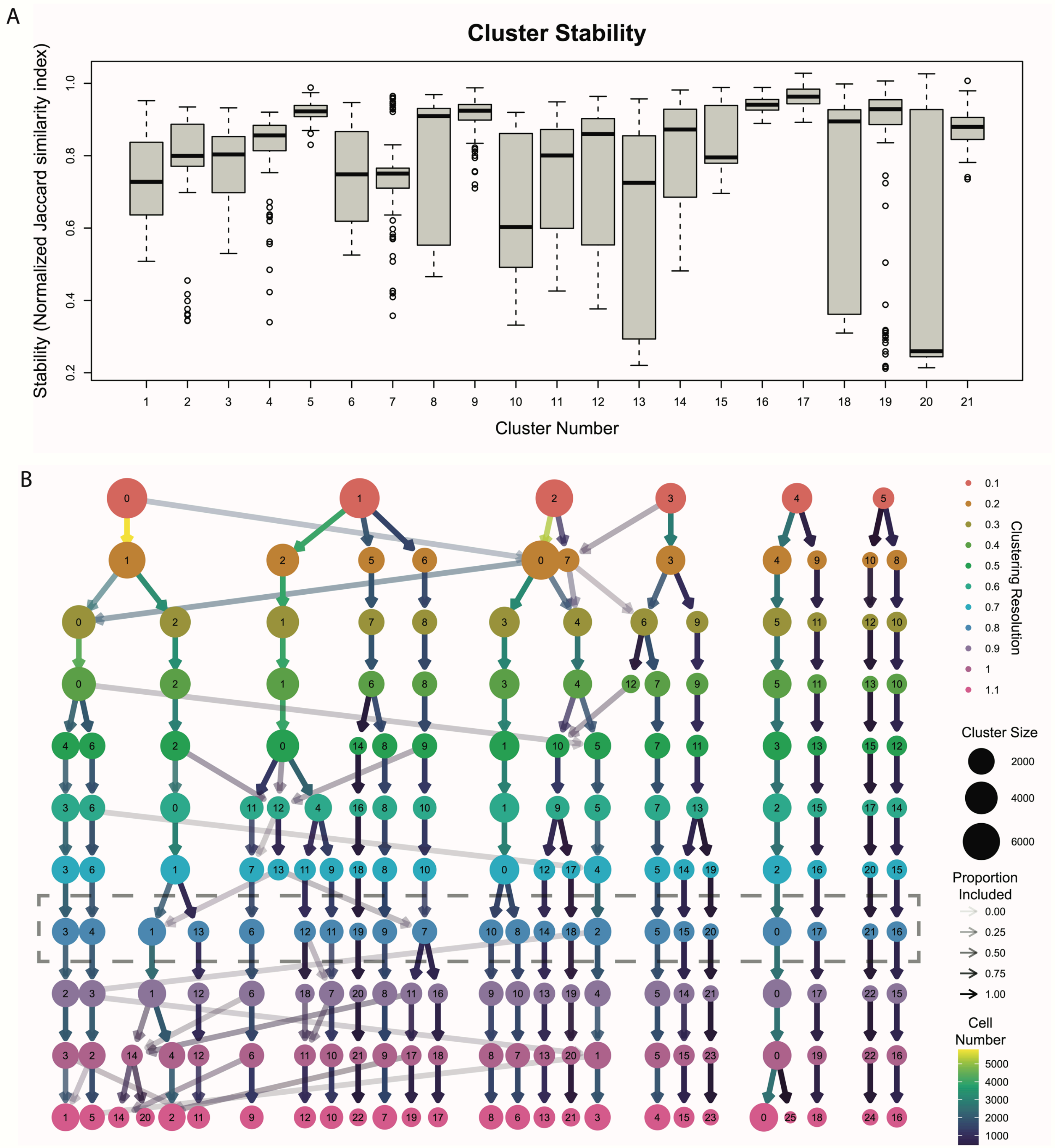

bioRxiv preprint doi: https://doi.org/10.1101/2024.06.06.597807; this version posted September 25, 2024. The copyright holder for this preprint (which was not certified by peer review) is the author/funder, who has granted bioRxiv a license to display the preprint in perpetuity. It is made available under aCC-BY 4.0 International license.

Figure S3

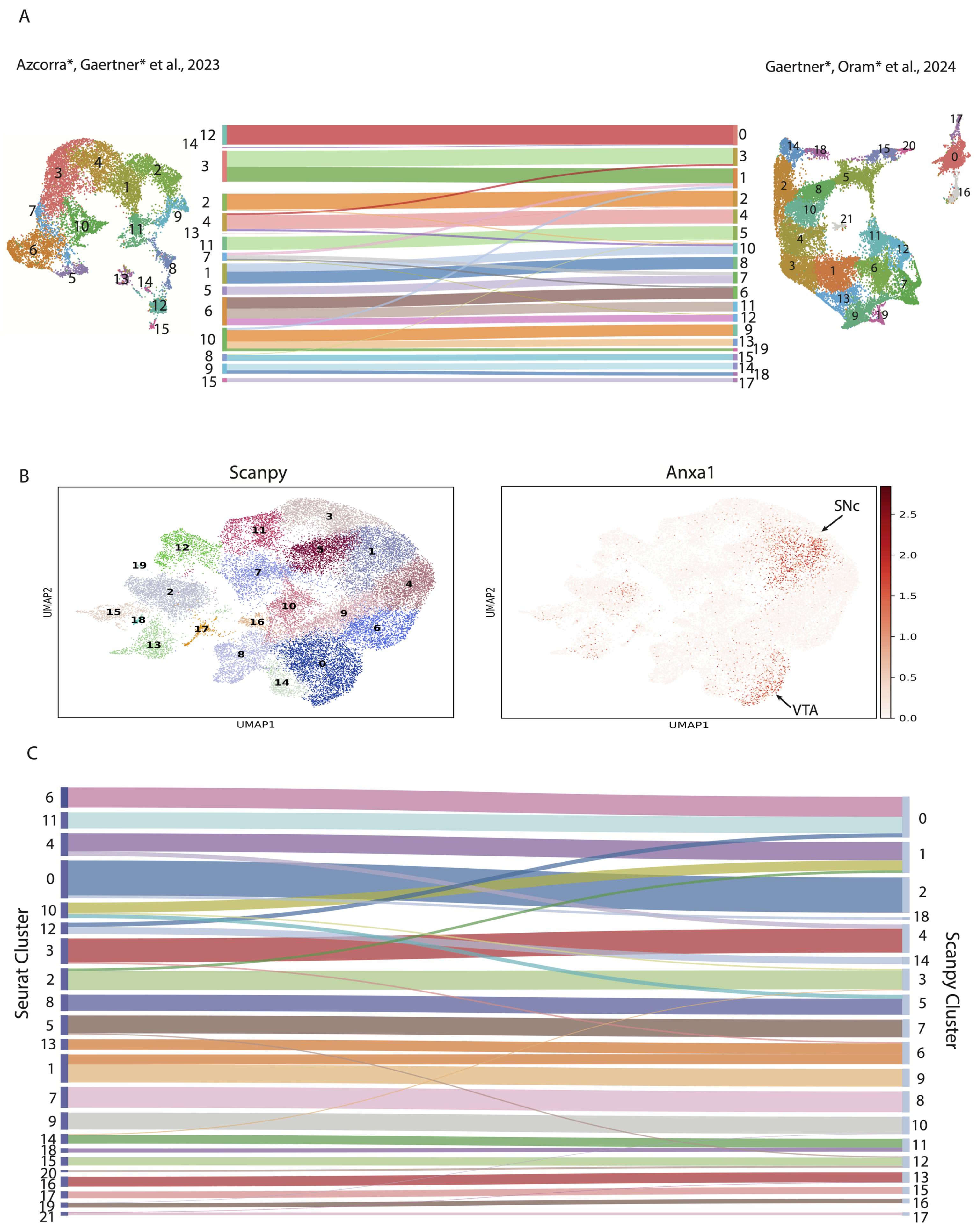

bioRxiv preprint doi: https://doi.org/10.1101/2024.06.06.597807; this version posted September 25, 2024. The copyright holder for this preprint (which was not certified by peer review) is the author/funder, who has granted bioRxiv a license to display the preprint in perpetuity. It is made available under aCC-BY 4.0 International license.

Figure S4

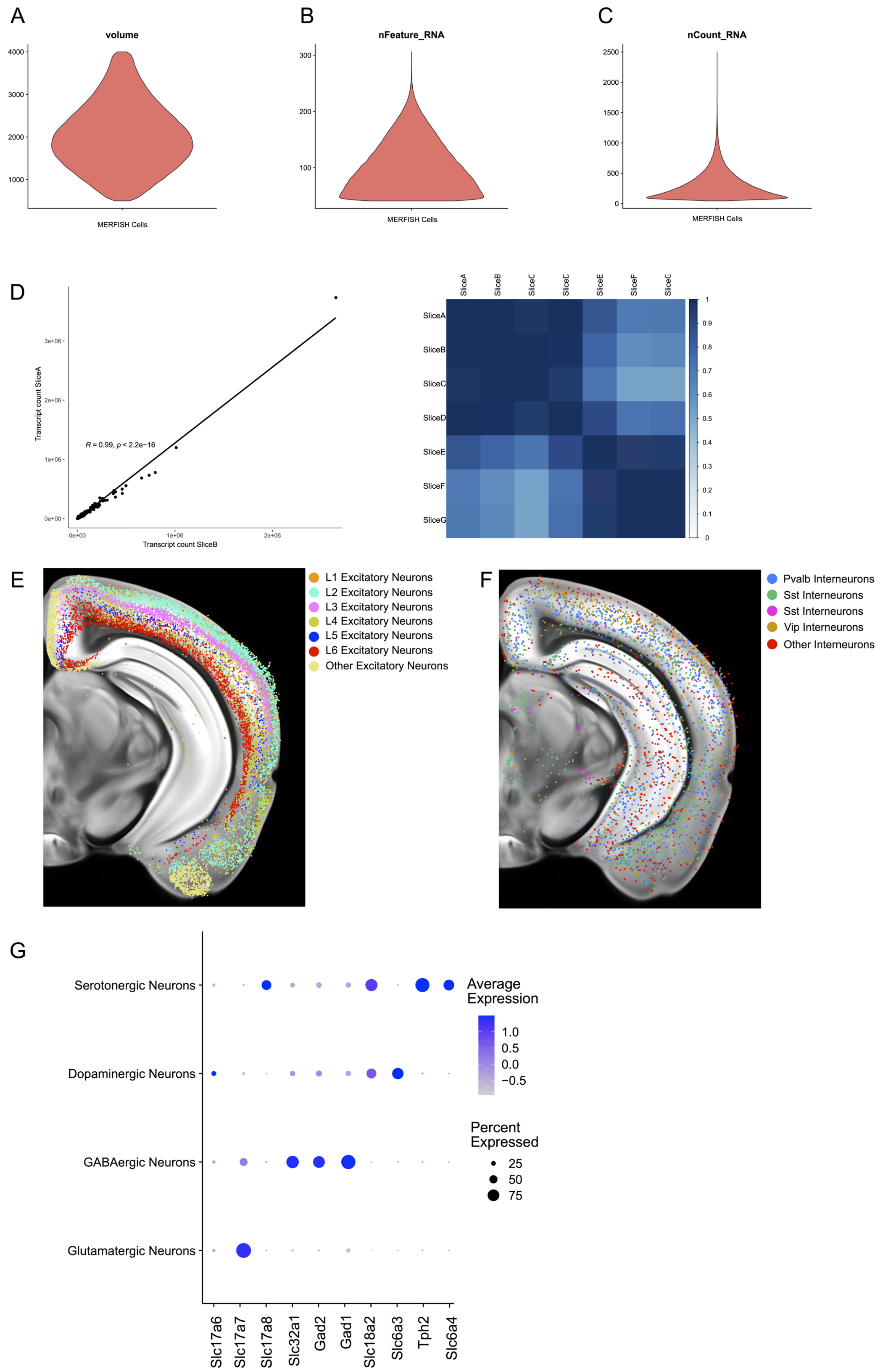

bioRxiv preprint doi: https://doi.org/10.1101/2024.06.06.597807; this version posted September 25, 2024. The copyright holder for this preprint (which was not certified by peer review) is the author/funder, who has granted bioRxiv a license to display the preprint in perpetuity. It is made available under aCC-BY 4.0 International license.

Figure S5

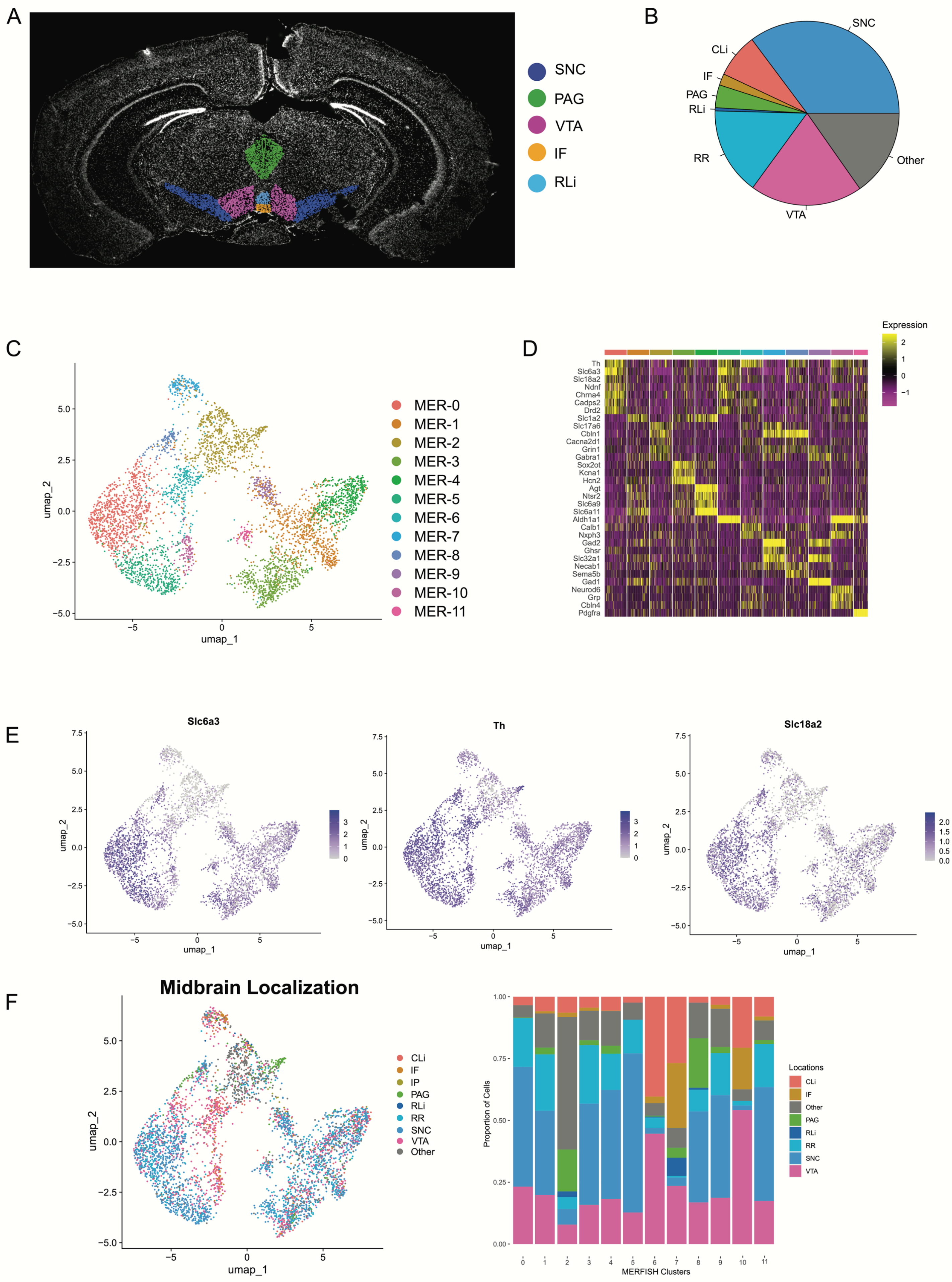

bioRxiv preprint doi: https://doi.org/10.1101/2024.06.06.597807; this version posted September 25, 2024. The copyright holder for this preprint (which was not certified by peer review) is the author/funder, who has granted bioRxiv a license to display the preprint in perpetuity. It is made available under aCC-BY 4.0 International license.

Figure S6

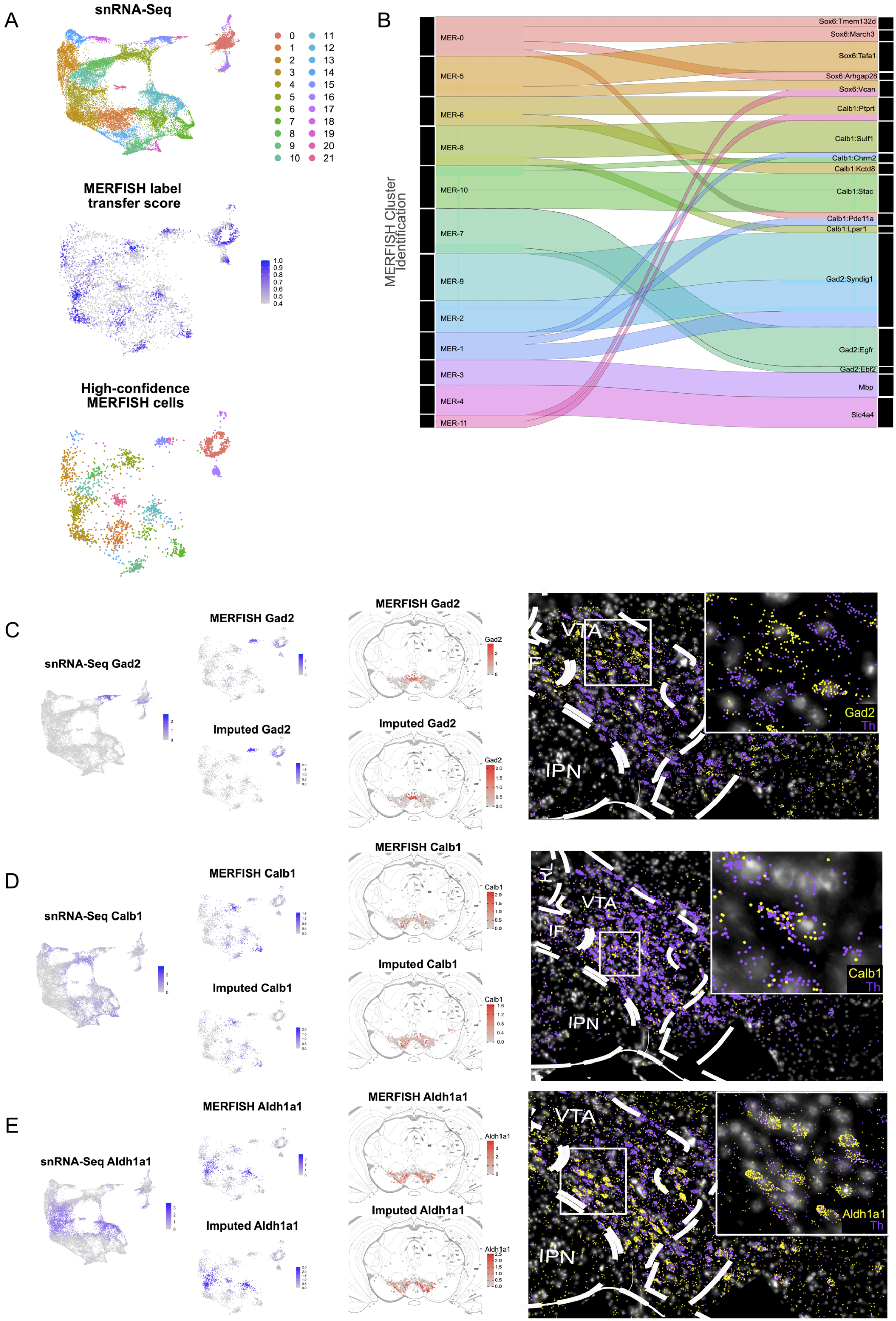

bioRxiv preprint doi: https://doi.org/10.1101/2024.06.06.597807; this version posted September 25, 2024. The copyright holder for this preprint (which was not certified by peer review) is the author/funder, who has granted bioRxiv a license to display the preprint in perpetuity. It is made available under aCC-BY 4.0 International license.

Figure S7

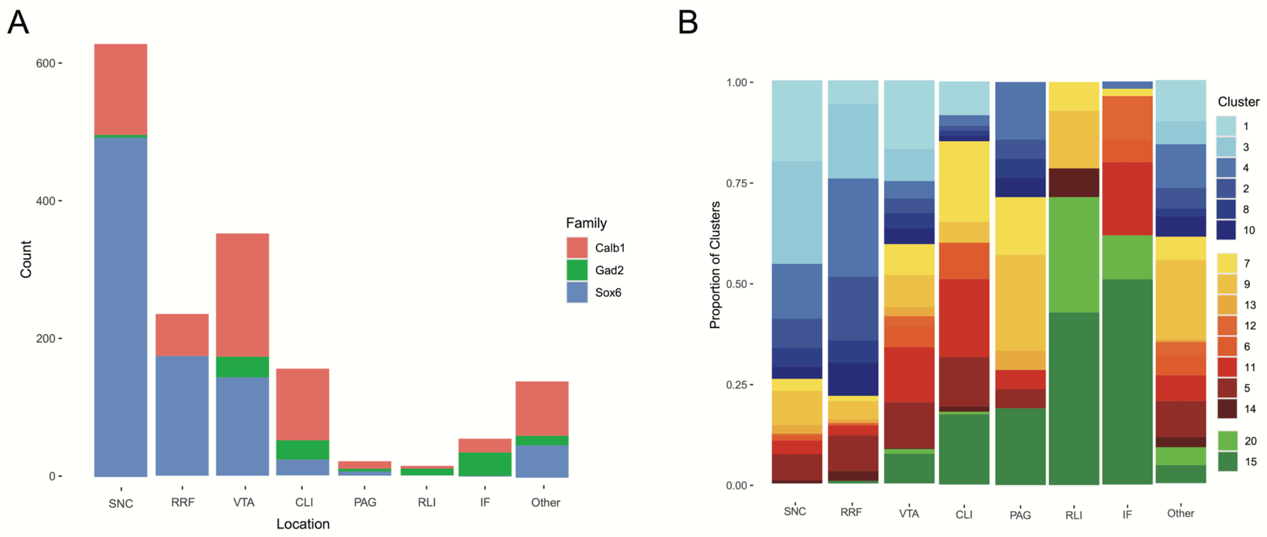

bioRxiv preprint doi: https://doi.org/10.1101/2024.06.06.597807; this version posted September 25, 2024. The copyright holder for this preprint (which was not certified by peer review) is the author/funder, who has granted bioRxiv a license to display the preprint in perpetuity. It is made available under aCC-BY 4.0 International license.

Figure S8

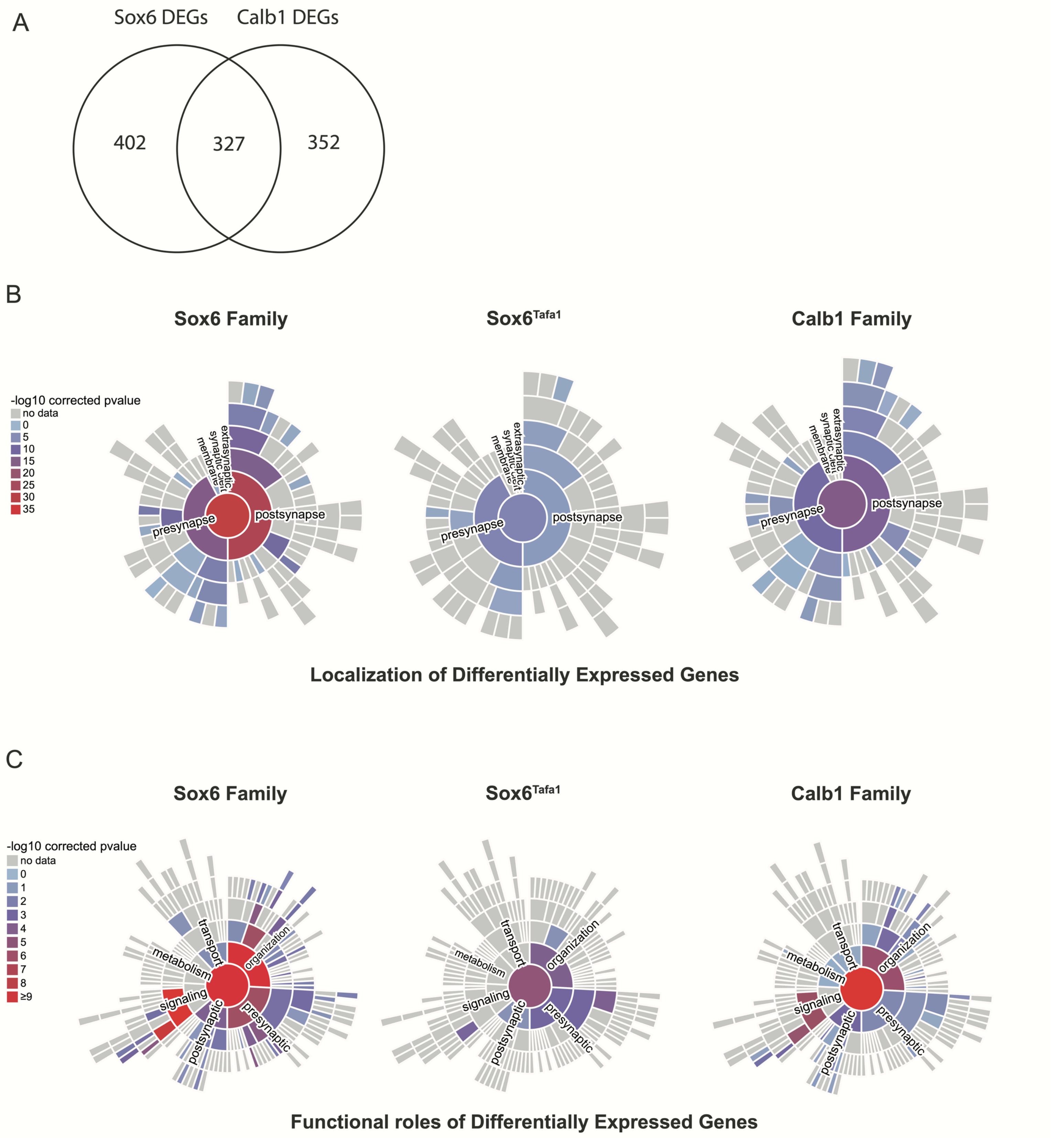

Supplement: Supplementary file 1 [file NIHPP2024.06.06.597807v3-supplement-1.pdf]
